# Commercialization of academic patents in Chinese universities: Antecedents and spatial spillovers

**DOI:** 10.1016/j.heliyon.2023.e14601

**Published:** 2023-03-15

**Authors:** Jiafeng Gu

**Affiliations:** Institute of Social Science Survey, Peking University, Beijing, 100871, China

**Keywords:** Commercialization of university patents, University innovation ecosystem, Basic research, Spatial spillover, China

## Abstract

The commercialization of academic patents is a basic means for universities to promote economic growth and upgrade the industrial innovation of enterprises. However, among developing countries, the commercialization rate of university patents is generally low. This study utilizes data from 65 universities which are directly under the Ministry of Education of China to analyze the influencing factors and mechanisms of academic patent commercialization. The findings show that the proportion of associate professors, the size of service staff transforming research and development achievement, and the proportion of basic research funding in universities are positively correlated with the commercialization rate of university patents. In addition, these factors indirectly affect the commercialization of university patents by affecting neighboring universities; that is, there are spatial spillover effects in the commercialization of university patents between neighboring universities. These empirical results indicate that universities can promote the commercialization of university patents by optimizing the structure of faculty, developing the R&D achievement transformation service staff team, and strengthening investment in basic research.

## 1 Introduction

In the context of technological innovation becoming an important driving force for economic and social development, innovation-driven economies have increasingly become the focus of international competition [1,2]. As the crucial force of knowledge innovation, universities shoulder an important historical mission in promoting technological innovation and leading the process of economic transformation [[3], [4], [5]]. Since 2010, universities in developed countries including the United States [6], Germany [7], the United Kingdom [8], Canada [9] have introduced various measures to optimize the allocation of patented technology commercialization resources, reshape the management mode of patent technology in universities, and actively promote the commercialization of university patents. However, in most developing countries, including China [10] and Brazil [11], there are some prominent problems in the commercialization of academic patents in universities. Therefore, the continuous improvement of the degree of commercialization of university patents in developing countries plays a very important role in continuously and steadily promoting regional economic and social development and is also an objective need for global economic development.

Commercialization of university patents is a process of converting academic patents from universities and industries into useful products or practical applications [12]. Commercialization of unviersity patents is an integral part of university intellectual property management [6]. China is the world's second largest economy, but the commercialization rate of Chinese university patents is only about 5% [13]. The low degree of university patents commercialization is also a universal challenge faced by most developing countries [14,15]. Since 2006, to build an innovative country, the Chinese government has allocated a sizable budget and spent a lot of money to support research and development (R&D) and university patents commercialization activities. At the same time, the Chinese government has vigorously promoted the commercialization strategy of university patents and has achieved good results [16]. Therefore, this study takes Chinese universities as the research object to discuss the influencing factors and impact paths of academic patent commercialization to provide beneficial experience sharing for developing countries. Specifically, based on a novel measure of patent value and using unique data on patent licensing in the U.S, Hsu et al. [17] examined university-level factors that could promote patent commercialization. This research aims to contribute to the literature by examining the evidence from China.

In addition, although there are many studies on the commercialization of academic patents, these studies generally ignore the impact of spatial proximity [12,18,19]. This type of research generally believes that neighboring universities are independent of each other in commercializing academic patents and are not affected by the context of their neighboring spatial characteristics [10,12,20]. This viewpoint can be classified as ‘‘spatial context-neutral’’ thesis. Unfortunately, the “spatial context-neutral” thesis has been questioned by some scholars. In contrast, an alternative popular viewpoint clearly asserts that the spatial contextual of universities and colleges affects academic patent commercialization [21]. For example, Spithoven et al. [22]'s research shows that distance affects university patents commercialization. The empirical evidence from Holm et al. [23] also shows the impact of distance on the commercialization of academic patents. This viewpoint can be classified as ‘‘spatial context-sensitive’’ thesis. This article hypothesizes that spatial context operationalized by the spatial weight matrix matters in commercializing academic patents [24]. To test this hypothesis, the strategic interaction between neighboring universities and colleges in the commercialization of Chinese university patents is examined. This means that the behavior of universities will not only directly affect university patents commercialization, but also indirectly affect the commercialization of university patents by affecting other neighboring universities. Therefore, this study will provide important opportunities for effective evaluations regarding the acute debate between two contrasting views: the spatial context-neutral thesis and the spatial context-sensitive thesis.

In order to reach these goals, this study attempts to answer the coming two questions:

Question 1: What factors will affect the commercialization of university patents?
Question 2: What is the impact pathway of these factors?

The answers to the above research questions can provide important theoretical support and practical guidance for the management and commercialization of patents of institutions such as universities, scientific researchers, enterprises, etc. The academic contributions of this paper are: First, the key antecedents of commercialization of university patents are identified and analyzed, which is helpful to promote effectively the transformation of academic scientific research achievements. Secondly, this research reveals the internal connection between basic academic research and university patents commercialization, which helps to correctly understand the important role and value of basic research in the commercialization of university patents. Thirdly, considering the spatial characteristics of university patents commercialization and the spatial interaction between neighboring universities, this paper takes advantage of spatial measurement methods as a way to study and confirm the existence of spatial effects.

## 2 Theoretical background and hypothesis development

University patents commercialization is an intricate and complex process involving the interests of universities, enterprises, and academic inventors. Therefore, many factors affect university patents commercialization [12]. Rahal and Rabelo [25] summarized the factors into five levels: institutional, inventor-related, technology-related, market and commercialization-related, and intellectual property-related factors. This classification is based on the summary and review of relevant literature before 2006. However, this classification is not complete. For example, factors related to spatial context are not included. In addition, different researchers select several of these factors for research based on their research goals and data availability. Wu, Welch and Huang [6] studied university patents commercialization based on individual and institutional factors. Gong and Peng [20] investigated the influence of patent policy factors on university patents commercialization. Burg, Du and Kers [19] explored the impact of organizational-, individual- and patent-level factors on the commercialization of university patents. Unfortunately, due to different research levels and different data, there is no consensus in the academic circles about the factors that affect and determine the commercialization of university patents, and even opposite conclusions have been reached. However, universities play a crucial role in the commercialization of academic patents, which is recognized by the academic community [5,16,26]. Therefore, it is important and pressing to study university patent commercialization based on the strategic arrangements of universities.

In addition, when studying the path to commercialization of academic patents, past research has focused on spillover effects from universities to enterprises [27]. When universities spill knowledge and innovation into enterprises, they often have certain spatial characteristics, which is the so-called spatial knowledge spillover [28]. Specifically, companies in neighboring universities are more likely to obtain spillover results from universities in innovation [29]. That is, in the commercialization of university patents, universities and neighboring companies are dependent on each other. However, past research in this area ignored the interdependence of neighboring universities in the commercialization of academic patents but assumed that neighboring universities are independent of each other. As a result, the spatial knowledge spillover mechanism from universities to neighboring universities has been ignored. In fact, neighboring universities are not independent of each other, and there are strategic interactions [30,31]. Therefore, it is natural to consider this so-called strategic interaction to reveal the specific mechanism of academic patent commercialization.

In universities, the academic rank of inventors often affects the commercialization of patents. The relationship between these two factors has aroused the attention of and caused controversy in the academic community. A study based on a sample of 5811 academic patents with US faculty as inventors shows that higher-ranked US scholars and academic researchers are lower probability to file their patents outside the university, which will reduce the probability of commercializing academic patents from universities [32]. Another study based on 736 academic individuals in 2006 showed that academic rank negatively affect external patenting [33]. In Italy, a study based on 3651 Italian academic patents showed that full professors’ patents are lower probability of being passed on to firms [34]. However, some studies support the opposite conclusion, suggesting that higher-ranked scholars and academic researchers are higher probability to realize the commercialization of academic patents. In Europe, academic inventors with a shorter time in their current job are less likely to assign the patent to the enterprise [35]. This view is also confirmed by empirical evidence from the United States [36] and the Netherlands [19].

In universities, the up-or-out promotion for faculty followed by life-long tenure intensifies the tenure competition of academic researchers, which urges lower-ranked academic researchers to prove the value of research by commercializing academic patents, thereby gaining an advantage in tenure competition [37]. Lower-ranked academic researchers generally lack research funding, and commercializing academic patents allows researchers to quickly obtain considerable research funding [38]. In addition, lower-ranked academic researchers are more likely to find entrepreneurial opportunities through the commercialization of academic patents [39]. In recent years, most well-known universities in China have already implemented the up-or-out policy. However, unlike the tenure track system of universities in western developed countries, the up-or-out system of Chinese universities generally emphasizes the publication of academic papers and other achievements, rather than patents [40]. Some Chinese universities have gradually realized this problem and have begun to adjust their up-or-out policies to include patent achievements in the evaluation criteria for professional titles. To a certain extent, this gives young researchers with lower professional titles an incentive to increase patent output and commercialization. Thus, it can be inferred that the higher the proportion of lower-ranked academic researchers in universities, the higher the degree of commercial transformation of university patents. Logically, the coming hypothesis is proposed.

Hypothesis 1(H1) The proportion of lower-ranked academic researchers in universities is positively affects university patents commercialization.

The commercialization of academic patents in universities is a complex process, from technological discovery to commercial application of enterprises [12]. This process involves three different subjects: academic scientists, universities and enterprises. Patent commercialization is a specialized market activity involving multiple links, such as searching for potential technology buyers, negotiation, and final contract signing [41]. Therefore, within universities, there needs to be corresponding personnel to assist academic scientists in university patents commercialization [42]. In this regard, the Technology Transfer Office (TTO) at the university plays a very important and irreplaceable role. After the university established a TTO, the commercialization of university patents improved [43,44].

In addition to establishing the Technology Transfer Office, universities generally recruit R&D achievement transformation service staff to promote university patents commercialization [45]. In maly universities and colleges, R&D achievement transformation service staff engages in social services, technical consultation, application, and promotion of academic patents in the process of transforming scientific research achievements of the university. Innovative universities need not only a group of outstanding researchers, but also a group of outstanding R&D achievement transformation service staff, which together constitute the ecosystems of innovation in universities [46]. In commercializing university patents, R&D achievement transformation service staff plays a key and crucial role in promoting the transfer and transformation of academic patents into real productivity [42]. In a university, the more R&D achievement transformation service staff, the greater the promotion of university patents, and the higher the degree of university patents commercialization.

Since the 1990s, Chinese universities have learned from western developed countries and established Technology Transfer Office one after another, hoping to promote the commercialization of university patents [47]. However, in practice, many Chinese universities still face the challenge of low technology transfer rate. Whether Technology Transfer Office effectively promotes the technology transfer and transformation of Chinese universities and what factors affect its role is still very controversial, and further research is still needed [48]. This situation is common in developing countries [49]. Compared with developed countries, the operating efficiency of Technology Transfer Office of universities in developing countries is lower, but it promotes university patents commercialization to a certain extent.

Therefore, the coming hypothesis is proposed.

Hypothesis 2(H2) The number of R&D achievement transformation service staff in universities is positively affects university patents commercialization.

The impact of basic research and applied R&D on university innovation and the commercialization of university patents is a hot concern of the academic community [50]. Some studies have shown that applied research in universities is helpful to the commercialization of university patents [51,52]. However, some scholars believe that basic research in universities has a significant and positive spillover effect on academic patent commercialization. For example, Thursby and Thursby [53] found that after the Bayh-Dole Act, the proportion of basic research funding in total R&D funding of American universities did not drop significantly. Existing empirical studies have shown that there is a significant and positive link between the research results of universities in basic and applied research [54,55]. There is a mutual promotion between basic university research and academic patent commercialization [56,57].

In developed countries, the sources of academic research funding for universities are diversified, and the proportion of industry funding is gradually increasing. For example, in the United Kingdom and Japan, industry funding for universities has increased significantly since 2004 [58]. In Germany, over the past decades, the share of industry-funded public research increased significantly, amounting to approximately 25% in 2007 [59]. However, in China, the research funding of universities is mainly from the government [60]. Among the universities which are directly under the Ministry of Education, the proportion of scientific research funding provided by the government is particularly high. These government-funded scientific research funds have been invested more in basic research of universities [61]. Basic research often takes a long time, and there are many uncertainties. Once a major breakthrough in basic research is made, it will bring about revolutionary changes [62]. In China, the government has increased its steady support for basic research in universities. Overall, this pattern will promote university patents commercialization.

According to these scholars, the coming hypothesis can be proposed.

Hypothesis 3(H3) The proportion of basic university research expenditures positively affects university patents commercialization.

The innovation activities of universities have obvious spatial spillover effects. An earlier study showed that from 1998 to 2002, in the United States and Canada, there were more than 2000 new inventions from technology spillovers of universities [26]. Multinational companies build R&D institutions near universities to make full use of the spatial spillover effect of universities and colleges in innovation [29]. The commercialization of university patents not only has spillover effects on neighboring companies but also on neighboring universities. In other words, there are the so-called strategic interactions between neighboring universities, and universities are dependent of each other [30,31]. A university promoted its patents commercialization by expanding the scale of lower-ranked academic researchers, increasing R&D achievement transformation service staff, and increasing investment in basic research. These practices will soon spill over to neighboring universities, making these universities learn and copy these practices through imitation. In this way, the so-called spatial lag effect appears [63]. Therefore, the following three assumptions were made.

Hypothesis 4(H4) The spatial lag of the proportion of lower-ranked academic researchers in universities positively affects university patents commercialization.

Hypothesis 5(H5) The spatial lag of the number of R&D achievement transformation service staff in universities positively affects university patents commercialization.

Hypothesis 6(H6) : The spatial lag of the proportion of university basic research expenditures positively affects university patents commercialization.

**Fig. 1:**
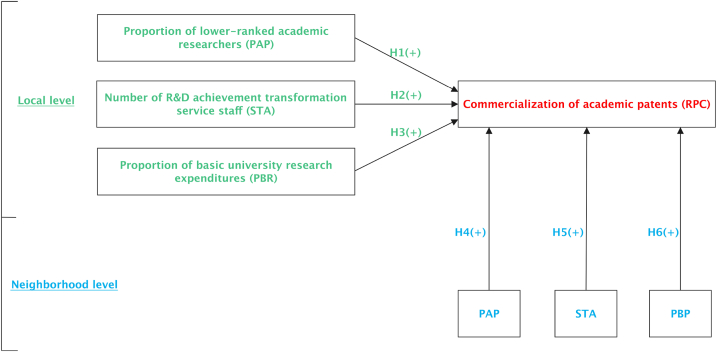
Theoretical model and related hypotheses.

The theoretical framework of this study is presented in Fig. 1. Antecedents' influence paths on university patents commercialization include direct influence paths and indirect influence paths. H1, H2 and H3 constitute the direct influence paths. H4, H5 and H6 and their corresponding dashed lines constitute the indirect influence paths. This study examines the link between the antecedents and university patents commercialization, which is based on the integration of local and neighborhood hierarchies.

## 3 Empirical strategy and methods

### 3.1 Data description

In order to control the influence of factors at the university level, this study uses universities which are directly under the Ministry of Education as the sample. Because all of these universities have been under the Ministry of Education, the sources of research funding and management systems are basically the same. Now, there are a total of 75 universities which are directly under the Ministry of Education. The “2017 Compilation of Science and Technology Statistics in Colleges and Universities” only reports relevant data from four kind of universities including science, engineering, agriculture, and medical universities, and does not report data from universities without engineering majors, such as language universities, universities of finance and economics, academies of fine arts, and conservatories of music. Therefore, these 10 universities which are directly under the Ministry of Education, including Beijing foreign language university, Beijing Language and Culture University, Beijing Broadcasting Institute, Central Conservatory of Music, Central Academy of Fine Arts, The Central Academy Of Drama, Shanghai University of Finance and Economics, Zhongnan University of Economics and Law, Central University of Finance and Economics, Foreign Economic and Trade University, were excluded from the research sample. Therefore, the samples used in this study are 65 universities directly under the Ministry of Education. Among these universities, 30 of them are “985″ universities and all of them are “211″ universities.

These universities are generally research and innovation universities or teaching and research universities in China, and most of them are involved in academic and applied research. According to the “2017 Compilation of Science and Technology Statistics in Colleges and Universities”, in 2016, the total number of universities in China counted in this data is 1,805, and these are active universities in scientific R&D activities. In other words, the number of colleges and universities which are directly under the Ministry of Education accounted for 3.43% of 1805 universities. It can be seen from the investment in scientific research funds that the total scientific research funds of the institutions directly under the Ministry of Education is 75.94841 billion yuan, while the total scientific research funds of 1805 universities are 153.71459 billion yuan. The former accounts for 49.41%. The commercialization income of academic patents was 560.715 million yuan, while for 1805 universities it was 1214.700 million yuan. The former accounted for 46.16% of the latter. These 65 universities which are directly under the Ministry of Education have led to the commercialization of Chinese universities’ patents [20]. Therefore, this study considers these 65 universities as the research subjects. The data comes from the “2017 Compilation of Science and Technology Statistics in Colleges and Universities”. If there are more recent data, such as the data of 2019, it is even better. However, the Ministry of Education no longer publishes scientific and technological statistics at the university level, but only publishes aggregated data. Therefore, it is impossible to conduct research from the university level using more recent data such as the data of 2019.

### 3.2 Variables and measurements

#### 3.2.1 Dependent variable

In terms of operational definitions, the commercialization rate of patents is a commonly used indicator for measuring the degree of patent commercialization conversion [18,20]. Here, the commercialization rate of university patents (RPCs) of a university is used as the dependent variable. The commercialization rate of university patents was obtained by calculating the percentage of the number of patents sold by the university to the number of patent applications of this university in 2016.

#### 3.2.2 Independent variables

This research mainly studies the influence of faculty composition, R&D achievement transformation service staff size, and basic research investment on university patents commercialization. In academic patent commercialization research, the position of academic inventors is a common method used to measure faculty composition [6,64]. In the operational definition of faculty composition, the proportion of associate professors (PAP) is used as the measurement indicator of the proportion of lower-ranked academic researchers. At the same time, the proportion of professors (PPR) is also introduced as a measurement indicator of the proportion of higher-ranked academic researchers. In the commercialization of academic patents, well-trained and professional R&D achievement transformation service staff plays a pivotal role [45]. In the operational definition of R&D achievement transformation service staff size, the number of R&D achievement transformation service staff (STA) is used as its measurement index. Meanwhile, the number of R&D faculty members (FAC) is introduced as a reference. Research funding is an important indicator of basic research investment [50]. Research activities in a general university can be divided into two categories: basic research and applied research. In the operational definition of basic research investment, the proportion of university basic research expenditures (PBR) is used as the measurement index [65]. This indicator is obtained by calculating the percentage of basic research expenditures for scientific research expenditures in the total research expenditure of universities. Meanwhile, the proportion of university applied research expenditures (PAR) was introduced as a reference [66].

In addition, there are two control variables: the proportion of invention patents (PIP) and the proportion of utility model patents (PUM) in patent applications [67,68]. This is to control for the possible impacts of patent types. The operational definitions of those variables are summarized in the following Table 1.

**Table 1:** Variable abbreviations and definitions.

Variable abbreviation	Variable	Variable definition
RPC	Rate of patent commercialization (%)	The number of patents sold/the number of patent applications
PAP	Proportion of associate professor（%）	The number of associate professor/the number of faculty
PPR	Proportion of professor（%）	The number of professor/the number of faculty
STA	Size of R&D service staff	The number of R&D achievement transformation service staff
FAC	Size of R&D faculty	The number of R&D faculty
PBR	Proportion of basic research (%)	The proportion of university basic research expenditures
PAR	Proportion of applied research (%)	The proportion of university applied research expenditures
PIP	Proportion of invention patents	The proportion of invention patents
PUP	Proportion of utility model patents	The proportion of utility model patents

Variable descriptions and correlations are reported in the coming Table 2.

**Table 2:** Variable descriptions and correlations.

Variable	M	S.D.	1	2	3	4	5	6	7	8	9
1.RPC	1.992	3.518	–								
2.PAP	36.065	5.585	0.313**	–							
3.PPR	28.963	7.686	0.173	0.251**	–						
4.STA	248.754	248.336	0.282**	0.078	0.156	–					
5.FAC	1462.369	1328.797	0.258*	0.244**	0.319***	0.505***	–				
6.PBR	41.508	19.893	0.267**	−0.006	0.209*	−0.012	0.233*	–			
7.PAR	43.990	20.744	−0.136	−0.108	−0.108	−0.031	−0.191	−0.617***	–		
8.PIP	0.767	0.204	0.212	0.419***	0.206*	0.032	0.166	0.0216*	−0.199***	–	
9.PUP	0.193	0.146	−0.163	−0.259**	−0.195	0.159	0.023	−0.084	−0.078	−0.55***	–

Note: *** p≤0.01.** p≤0.05.* p≤0.1.

In addition, geographical information including the latitude and longitude of each university are also needed to determine the spatial location of each university.

### 3.3 Analysis method

Referring to previous studies [69,70], the spatial lag model is used in this study. The three specific models are as follows:

(1) $${RPC}_{i}=C+\beta_{1}{PAP}_{i}+\rho W\times {PAP}_{i}+\beta_{2}{PPR}_{i}+\beta_{3}{STA}_{i}+\beta_{4}{FAC}_{i}+\beta_{5}{PBR}_{i}+\beta_{6}{PAR}_{i}+\beta_{7}{PIP}_{i}+\beta_{8}{PUP}_{i}+\varepsilon_{i},\varepsilon_{i}∼N\left(0,\sigma^{2}\right)\;i=1,2,\ldots \ldots ,65$$

In formula (1), the spatial lag of PAP (W×PAP) is one of the key independent variables.

(2) $${RPC}_{i}=C+\beta_{1}{PAP}_{i}+\beta_{2}{PPR}_{i}+\beta_{3}{STA}_{i}+\rho W\times {STA}_{i}+\beta_{4}{FAC}_{i}+\beta_{5}{PBR}_{i}+\beta_{6}{PAR}_{i}+\beta_{7}{PIP}_{i}+\beta_{8}{PUP}_{i}+\varepsilon_{i},\varepsilon_{i}∼N\left(0,\sigma^{2}\right)\;i=1,2,\ldots \ldots ,65$$

In formula (2), the spatial lag of the STA (W×STA) is also one of the key independent variables.

(3) $${RPC}_{i}=C+\beta_{1}{PAP}_{i}+\beta_{2}{PPR}_{i}+\beta_{3}{STA}_{i}+\beta_{4}{FAC}_{i}+\beta_{5}{PBR}_{i}+\rho W\times {PBR}_{i}+\beta_{6}{PAR}_{i}+\beta_{7}{PIP}_{i}+\beta_{8}{PUP}_{i}+\varepsilon_{i},\varepsilon_{i}∼N\left(0,\sigma^{2}\right)\;i=1,2,\ldots \ldots ,65$$

This is the Spatial Lag Model (SLM). The spatial lag term of the traditional SLM is the spatial lag variable of the dependent variable, while the spatial lag variable of the independent variables is used in this study, sequentially. In the above formula, *W* is the spatial weight matrix which contains information about the proximity of any two universities to each other. Here, it's the spatial inverse distance weight matrix. In econometrics, the estimate of ρ is often used to determine whether there is a spatial spillover effect [69,70].

## 4 Results

### 4.1 The antecedents and spillover of commercialization of academic patents

The empirical results are reported and summarized in the coming Table 3. Models 1, 2, and 3 in Table 1 correspond to (1), (2), (3), respectively.

**Table 3:** Results of the baseline models.

	Model 1	Model 2	Model 3
PAP	0.173**(2.32)	0.17**(2.3)	0.17**(2.29)
PPR	−0.043 (-0.82)	−0.041 (-0.79)	−0.042 (-0.8)
STA	0.004**(2.22)	0.004**(2.28)	0.004**(2.22)
FAC	0.001 (1.25)	0.001 (1.31)	0.001 (1.28)
PBR	0.053**(2.17)	0.053**(2.21)	0.053**(2.17)
PAR	0.001 (0.01)	−0.001 (-0.05)	−0.001 (-0.02)
PIP	−1.478 (-0.63)	−1.534 (-0.66)	−1.425 (-0.61)
PUP	−4.243 (-1.34)	−4.232 (-1.34)	−4.222 (-1.33)
_cons	−5.399 (-1.38)	−5.464 (-1.41)	−5.374 (-1.37)
ρ			
W×PAP	0.07**(2.34)		
W×STA		0.019**(2.55)	
W×PBR			0.073**(2.34)
Wald $\chi^{2}$	31.81***	33.21***	31.8***
Pseudo $R^{2}$	0.329	0.338	0.329
Wald test of spatial terms	5.47**	6.49**	5.47**

Note: *** p≤0.01. ** p≤0.05. T value in brackets.

As shown in Table 3, all the coefficients of PAP in three models are all positive, which is significant at the 0.05 level. It shows that in a university, the higher the proportion of associate professors, the higher the commercialization rate of university patents. Thus, Hypothesis 1 is confirmed. In contrast, the coefficients of PPR are all not significant in three models. It shows that the proportion of professors has no significant impact on the commercialization rate of university patents. It further shows that associate professors will have greater motivation in the process of promoting university patent commercialization because it can effectively illustrate the value of academic research, which is very beneficial for their promotion to professors.

In Table 3, all the coefficients of the STA in three models are positive. This shows that the higher the number of R&D achievement transformation service staff, the stronger the promotion and promotion of academic patent commercialization, and the higher the commercialization rate of university patents. Thus, Hypothesis 2 s confirmed. In contrast, the coefficients of FAC are not significant, which shows that the number of R&D faculties has no significant impact on the commercialization rate of academic patents in universities. R&D faculty are generally good at doing research but are not good at commercial promotion and transformation of university patents. This further illustrates the indispensable bridge role played by R&D achievement transformation service staff to connect academic inventors and enterprises in the process of university patent commercialization.

In Table 3, all the coefficients of PBR in the three models are positive, which shows that the higher the proportion of scientific research funds invested by universities in basic research, the higher the commercialization rate of university patents. Thus, Hypothesis 3 is confirmed. In contrast, the coefficients for PAR are not significant, which shows that the proportion of scientific research funds invested by universities in applied research has no significant impact on the commercialization rate of university patents. The degree of commercialization of academic patents is often positively related to the quality and value of university patents. It means that universities and colleges intensify basic research and are more likely to produce high-value academic patents, which will promote the commercialization of academic patents.

The coefficients for PIP and PUP are not significant. This shows that the types of academic patents applied by universities do not affect the commercialization rate of university patents.

### 4.2 Spatial spillover effect

To test whether there is a so-called spatial spillover in the commercialization of university patents by neighboring universities and colleges, one of the more common methods is to estimate ρ. According to the procedures of Anselin, Florax and Rey [70], the estimate of ρ is presented in Table 3. In Model 1, the estimate of ρ is 0.07, which is statistically significant at the 0.05 level. Thus, Hypothesis 4 is confirmed. When the proportion of lower-ranked academic researchers in a university is relatively high, which inspires the motivation of lower-ranked academic researchers to accelerate the commercialization of patents, neighboring universities will follow this approach and expand the size of lower-ranked academic researchers to increase the commercialization rate of university patents. In Model 2, the estimate of ρ is 0.019, which is statistically significant at the 0.05 level. Thus, Hypothesis 5 is confirmed. When a university accelerates the professional services of patent commercialization by expanding the size of R&D achievement transformation service staff, neighboring universities will copy this practice and recruit more R&D achievement transformation service staff to increase the commercialization rate of university patents. In Model 3, the estimate of ρ is 0.073, which is statistically significant at the 0.05 level. Thus, Hypothesis 6 is confirmed. When a university raises the rate of commercialization of academic patents by expanding its funding for basic research to produce high-quality patents, neighboring universities will also learn from this practice and increase funding for basic research to increase the commercialization rate of university patents.

**Fig. 2:**
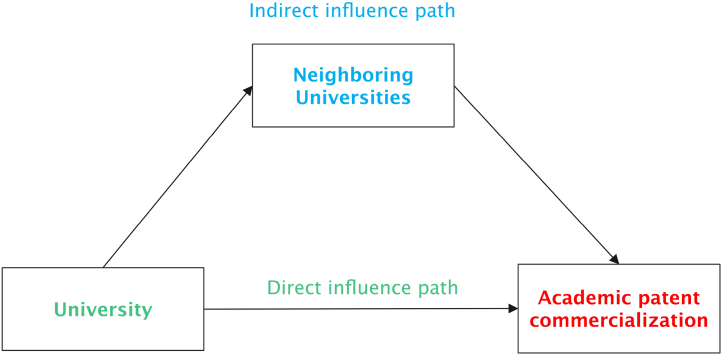
The dual-path influence mechanism.

According to Table 3, after introducing the spatial lag of the target variables to estimate ρ, the regression coefficients are all positive and all are statistically significant, which shows that the effective practices of neighboring universities in the process of promoting university patents commercialization have obvious positive spatial spillover benefits. Once the practice of promoting university patents commercialization is proven to be effective, neighboring universities will follow up and adopt similar practices to promote the degree of commercialization of university patents. It can be seen that in the process of promoting university patents commercialization, neighboring universities are not independent of each other, but are interconnected. In other words, neighboring universities have strategic interactions in promoting university patents commercialization [30,31]. This also shows that the solid path and the dashed path shown in Fig. 1 are both true. In other words, universities not only have a direct impact on university patents commercialization through their own investment, but also have an indirect impact on the commercialization of academic patents by influencing the input of other neighboring universities. This dual-path influence mechanism is summarized in Fig. 2. Thus, this research shows that spatial context really matters in commercializing academic patents. This well supports the ‘‘spatial context-sensitive’’ thesis [22,23].

### 4.3 Robustness test

Although the spillover effect models of academic patent commercialization in universities have been confirmed in empirical tests, once the measurement method of university patents commercialization changes, or the definition of the relationship between neighboring universities changes, whether the influence of relevant factors on the commercialization of university patents is still valid, further verification is needed [71,72]. In other words, whether the explanatory power of the commercialization rate of university patents can be maintained, a robustness test is required.

To test the robustness of the determinants of the commercialization rate of university patents and the so-called spatial spillover effect between neighboring universities, it is necessary to implement two rounds of robustness tests. The first is to change the measurement of academic commercialization rates. Here, the number of patents sold is divided by the number of patent authorizations to measure the commercialization rate of university patents. The dependent variable is named RPC1. The core regression results are summarized in Models 4, 5, and 6 in Table 4.

**Table 4:** RPC1 as the explained variable.

	Model 4	Model 5	Model 6
PAP	0.241**(2.43)	0.237**(2.42)	0.237**(2.39)
PPR	−0.075 (-1.08)	−0.073 (-1.06)	−0.074 (-1.06)
STA	0.005**(2.32)	0.006**(2.41)	0.005**(2.32)
FAC	0.001 (1.16)	0.001 (1.23)	0.001 (1.19)
PBR	0.066**(2.02)	0.066**(2.08)	0.065**(2.02)
PAR	0.003 (0.08)	−0.005 (-0.17)	0.004 (0.11)
PIP	−1.379 (-0.44)	−1.494 (-0.48)	−1.306 (-0.42)
PUP	−6.629 (-1.57)	−6.649 (-1.59)	−6.605 (-1.56)
_cons	−6.719 (-1.29)	−6.782 (-1.32)	−6.673 (-1.28)
ρ			
W×PAP	0.105***(2.65)		
W×STA		0.029***(2.97)	
W×PBR			0.111***(2.67)
Wald $\chi^{2}$	33.92***	36.41***	34.03***
Pseudo $R^{2}$	0.343	0.359	0.344
Wald test of spatial terms	7.03**	8.85**	7.11**

Note: *** p≤0.01. ** p≤0.05. T value in brackets.

Second, the spatial weight matrix is changed to test the influence of the relationship between different spatial positions on the regression results. Here, the previously used spatial inverse distance matrix (W) is replaced by the newly constructed spatial inverse-distance contiguity matrix (M) [73,74]. The empirical results are reported separately in Model 7, Model 8 and Model 9 of Table 5.

**Table 5:** Spatial regression models with a different spatial matrix.

	Model 7	Model 8	Model 9
PAP	0.168**(2.24)	0.165**(2.24)	0.165**(2.2)
PPR	−0.03 (-0.75)	−0.037 (-0.71)	−0.038 (-0.72)
STA	0.004**(2.17)	0.004**(2.23)	0.004**(2.13)
FAC	0.001 (1.25)	0.001 (1.31)	0.001 (1.27)
PBR	0.054**(2.22)	0.054**(2.24)	0.054**(2.2)
PAR	0.002 (0.07)	−0.003 (-0.13)	0.003 (0.13)
PIP	−1.43 (-0.6)	−1.491 (-0.64)	−1.269 (-0.53)
PUP	−4.285 (-1.34)	−4.306 (-1.37)	−4.096 (-1.28)
_cons	−5.304 (-1.35)	−5.198 (-1.34)	−5.453 (-1.38)
ρ			
W×PAP	0.07**(2.34)		
W×STA		0.021***(2.63)	
W×PBR			0.073**(2.04)
Wald $\chi^{2}$	30.9***	33.76***	30.01***
Pseudo $R^{2}$	0.322	0.342	0.316
Wald test of spatial terms	4.81**	6.89**	4.16**

Note: *** p≤0.01. ** p≤0.05. T value in brackets.

Table 4, Table 5 yield almost the same results as Table 2. In addition, these core independent variables have significant positive spatial spillover benefits. This shows that in the case of different measurements of academic patent commercialization rate or different spatial weights, the impact of these core independent variables on the commercialization of university patents and their spatial spillover benefits remains unchanged.

Finally, considering the special characteristics of Beijing and Shanghai in higher education, universities located in these two places are excluded from the sample universities for robustness testing, and the results are summarized in Table 6. It is found that the empirical results do not change by excluding the universities located in Beijing and Shanghai.

**Table 6:** Spatial regression models without universities in Beijing and Shanghai.

	Model 10	Model 11	Model 12
PAP	0.187**(2.5)	0.184**(2.49)	0.184**(2.46)
PPR	−0.027 (-0.5)	−0.025 (-0.47)	−0.026 (-0.49)
STA	0.005**(2.35)	0.005**(2.41)	0.005**(2.35)
FAC	0.001 (1.05)	0.001 (1.11)	0.001 (1.08)
PBR	0.005**(2.32)	0.06**(2.37)	0.059**(2.32)
PAR	0.001 (0.06)	−0.001 (-0.01)	0.001 (0.03)
PIP	−2.002 (-0.85)	−2.062 (-0.89)	−1.951 (-0.83)
PUP	−4.093 (-1.28)	−4.076 (-1.29)	−4.069 (-1.28)
_cons	−6.45 (-1.65)	−6.518 (-1.68)	−6.426 (-1.64)
ρ			
W×PAP	0.074**(2.49)		
W×STA		0.019***(2.71)	
W×PBR			0.077**(2.49)
Wald $\chi^{2}$	31.7***	33.25***	31.67***
Pseudo $R^{2}$	0.35	0.36	0.349
Wald test of spatial terms	6.21**	7.32**	6.19**

Note: *** p≤0.01. ** p≤0.05. T value in brackets.

## 5 Discussion

### 5.1 Theoretical implications

The commercialization of university patents is a key and crucial process for the realization of the value of academic patents and then bringing real value to universities, enterprises, and society. This research explores the antecedents and spatial spillovers of the commercialization of academic majors. The theoretical contributions of this research are reflected in the following four aspects.

First, this research systematically explores the factors influencing academic patent commercialization in universities, which provides important and convincing empirical evidence for resolving academic disputes. Existing research has drawn inconsistent conclusions when studying the factors affecting university patents commercialization, and even conflicts with each other. For example, it's hot debated about the influence of academic inventors' positions on the commercialization of academic patents. Some studies have found that higher-ranked academic researchers are less likely to implement the commercialization of academic patents [[32], [33], [34]]. These can be regarded as opponents of higher-ranked academics. However, some studies support the opposite conclusion, suggesting that higher-ranked academics are more likely to realize university patents commercialization [19,35,36]. These can be regarded as supporters of higher-ranked academics. This study found that the proportion of associate professors has a positive correlation with the commercialization rate of academic patents in this university, but the proportion of professors has no significant impact on the commercialization rate of university patents. It can be seen that the empirical evidence of this study strongly supports the view of opponents of higher-ranked academics [[32], [33], [34]]. It can be seen that, compared with higher-ranked academics, lower-ranked academics have shown greater aggressiveness in the commercialization of academic patents, mainly because of fierce tenure competition [37], obtaining external funds [38] and pursuing possible entrepreneurial opportunities [39].

Second, this research investigates the impact of R&D achievement transformation service staff on university patents commercialization and identify the direct effect path and indirect effect path, which enriches the research on the commercialization model and behavior of university and academic patents. Existing research on the commercialization of university patents is mainly limited to discussing whether the university R&D faculty is sufficient, while ignoring the role of R&D achievement transformation service staff in the university innovation ecosystem [46]. This study takes size of R&D achievement transformation service staff as an important variable, deeply analyzes its influence on the commercialization of academic patents, and expands the research on academic commercialization of universities. This study finds that the size of R&D achievement transformation service staff positively affects the academic patent commercialization rate, and the number of R&D faculty has no significant influence on the commercialization rate of academic patents in universities. This study emphasizes the important and indispensable role of R&D achievement transformation service staff in the innovation ecosystem of universities, and is an important and crucial force in promoting the commercialization of academic patents in universities, which effectively expands the previous related research [42,45].

Third, this research reveals the internal connection between basic research in universities and the commercialization of university patents and helps to correctly understand the important role and value of basic research in the commercialization of academic patents. Since the US government passed the Bayh-Dole Act in the winter of 1980, university patents commercialization has developed rapidly. At the same time, some scholars worry that commercialization of university patents may negatively affect basic research in universities [50]. Universities may give up or weaken their investment in basic research for short-term benefits [75]. This study shows that such concerns do not have a solid foundation. Our results show that the proportion of basic research funding in universities positively affects the commercialization rate of university patents, while the proportion of applied research funding is not related to the commercialization rate of university patents. This shows the importance of basic research scientists in university innovation [76]. Major technological changes often originate from basic research [77]. Similar views have also been reported in other studies [53,54,56]. In addition to providing important empirical evidence for these views, this research provides an important theoretical basis for universities to further strengthen their investment in basic research. Universities have rapidly developed research fields with rich commercial potential, but the research that produced them is essentially basic research [78]. The empirical evidence in this study supports their judgment.

Finally, this research reveals and verifies the strategic interaction and spatial spillover of universities in the commercialization of university patents. Due to reasons such as analysis level and data acquisition, existing studies have assumed that universities are independent of each other in the commercialization of university patents, and there is no strategic interaction between neighboring universities [12,79]. This article discusses the relationship between neighboring universities in the commercialization of academic patents, and finds that the practices of universities, such as increasing the proportion of lower-ranked academics, raising the number of R&D achievement transformation service staff, and increasing the proportion of basic research funding, have a significant spatial lag effect and will spill over to neighboring universities and colleges and affect university patents commercialization. This confirms that there are different forms of strategic interactions between neighboring universities in the commercialization of academic patents, and they are mutually dependent, rather than independent. This kind of interdependence and strategic interaction between neighboring universities also exists in college admissions competition and other activities [30,31]. In addition, past research has emphasized the spillover effect of universities on the technological innovation of neighboring enterprises, while ignoring the spillover effect of universities to other neighboring universities [26,29]. This research fills this gap, confirms the so-called spatial spillover of university patent commercialization among neighboring universities, and deepens the understanding of the mechanism and path of academic patent commercialization of universities.

### 5.2 Practical implications

The results of this study have important implications for social practice. First, in the science and technology evaluation system of universities, researchers in lower-ranked academics would be encouraged to participate in the commercialization of academic patents, if patent commercialization becomes one of the criteria for promotion of professional titles. Therefore, universities can incorporate patent output and patent commercialization into the criteria for promotion of professional titles. Second, from the height of the construction of the university's innovation ecosystem, universities should pay attention to the bridge role of R&D achievement transformation service staff in the commercialization of academic patents, and continuously develop a team of R&D achievement transformation service staff with professional capabilities. Third, universities need to continuously increase their investment in basic research, occupy the commanding heights of technological innovation, and grasp the initiative in the commercialization of academic patents. Finally, it is necessary to make use of the resources and experience sharing in innovation and academic patent commercialization between neighboring universities, establish a mutual assistance mechanism, and jointly promote university patents commercialization.

As an integral part of the collaborative development of regional innovation, the regional university academic patent commercialization alliance is an inevitable choice to promote regional higher education to adapt to the national innovation development strategy, and has irreplaceable strategic significance to the improvement of the academic patent transformation capacity of regional higher education. To explore the optimal strategy for the development of regional universities' academic patent commercialization alliance, the government, as a macro regulator, needs to do a good job of top-level policy design and coordination; while universities, as participants, should set the right motives, take the initiative and actively engage in such alliances.

## 6 Conclusion

This study uses a sample of 65 universities which are directly under the Ministry of Education of China in 2016 to examine the factors affecting the commercialization of university patents and the so-called spatial spillover using the spatial lag model. This study shows that faculty composition, the size of R&D achievement transformation service staff, and basic research investment affect the commercialization of university patents. In addition, this study also shows that neighboring universities are interdependent in commercializing university patents, that is, there is a spatial spillover effect. Neighboring universities can enhance innovation and academic patent commercialization competency.

This study has some limitations, and further research is needed in the future. First, it uses cross-sectional data. Thus, it mainly focuses on research on correlation, not the research on causality. If the data is available, panel data can be used for causality testing in the future. If the cross-sectional data is continued, the current sample size is relatively small and can be further expanded in the future to improve the accuracy of regression analysis. Second, this research focuses on the spatial spillover effects between neighboring universities, which can be extended to neighboring universities and neighboring enterprises in the future. In this way, the spatial spillover effects of university innovation will be more comprehensive. Third, this study focused on Chinese universities. If relevant data from abroad are available, international comparison will be an important direction for future research, and the research can be extended to developing countries or emerging economies. In addition, this study has no variables on the quality of university patents, and the impact of university patent quality can be studied in the future. Finally, one more note is needed. The intensity of industry-university collaboration (IUC) can be another important factor in the commercialization of university [[80], [81], [82]]. Due to data limitations, the impact of the intensity of IUC is not explored here. It will be necessary to study the impact of this variable on the commercialization of university patents when data are available.

## Ethical statement

This research is funded by the Social Science Foundation of China (17BSH122). The authors declare that they have no conflict of interest. Because the data in this research is not collected from human subjects and is not involving Human Participants and/or Animals, EA is no needed in this research.

## Funding

The author(s) disclosed receipt of the following financial support for the research, authorship, and/or publication of this article: This research is funded by the National Social Science Funds of China (17BSH122).

## Author contribution statement

Jiafeng Gu: Conceived and designed the experiments; Performed the experiments; Analyzed and interpreted the data; Contributed reagents, materials, analysis tools or data; Wrote the paper.

## Data availability statement

Data will be made available on request.

## Declaration of competing interest

The author(s) declared no potential conflicts of interest with respect to the research, authorship, and/or publication of this article.
